# Roles of Caloric Restriction, Ketogenic Diet and Intermittent Fasting during Initiation, Progression and Metastasis of Cancer in Animal Models: A Systematic Review and Meta-Analysis

**DOI:** 10.1371/journal.pone.0115147

**Published:** 2014-12-11

**Authors:** Mengmeng Lv, Xingya Zhu, Hao Wang, Feng Wang, Wenxian Guan

**Affiliations:** 1 Department of General Surgery, Nanjing Medical University Affiliated Cancer Hospital, Cancer Institute of Jiangsu Province, Nanjing, China; 2 The First Clinical School of Nanjing Medical University, Nanjing, China; 3 Gulou Clinical Medical College, Nanjing Medical University, Nanjing, China; 4 Department of Gastrointestinal Surgery, Nanjing Gulou Hospital Affiliated to Medical College of Nanjing University, Nanjing, China; Universidad Pablo de Olavide, Centro Andaluz de Biología del Desarrollo-CSIC, Spain

## Abstract

**Background:**

The role of dietary restriction regimens such as caloric restriction, ketogenic diet and intermittent fasting in development of cancers has been detected via abundant preclinical experiments. However, the conclusions are controversial. We aim to review the relevant animal studies systematically and provide assistance for further clinical studies.

**Methods:**

Literatures on associations between dietary restriction and cancer published in PubMed in recent twenty years were comprehensively searched. Animal model, tumor type, feeding regimen, study length, sample size, major outcome, conclusion, quality assessment score and the interferential step of cancer were extracted from each eligible study. We analyzed the tumor incidence rates from 21 studies about caloric restriction.

**Results:**

Fifty-nine studies were involved in our system review. The involved studies explored roles of dietary restriction during initiation, progression and metastasis of cancer. About 90.9% of the relevant studies showed that caloric restriction plays an anti-cancer role, with the pooled OR (95%CI) of 0.20 (0.12, 0.34) relative to controls. Ketogenic diet was also positively associated with cancer, which was indicated by eight of the nine studies. However, 37.5% of the related studies obtained a negative conclusion that intermittent fasting was not significantly preventive against cancer.

**Conclusions:**

Caloric restriction and ketogenic diet are effective against cancer in animal experiments while the role of intermittent fasting is doubtful and still needs exploration. More clinical experiments are needed and more suitable patterns for humans should be investigated.

## Introduction

Cancer was the second leading cause of mortality worldwide and its incidence has been increasing during the last decades [1], [2]. Epidemiological studies report that diet plays an important role in the initiation, promotion and progression of common cancers [3]. For centuries, dietary restriction has been widely recognized with health benefits and consistently been shown to extend lifespan in various mammals [4], [5]. Its anticancer effects have recently been identified via numerous animal experiments. Among various dietary restriction regimens, caloric restriction (CR), intermittent fasting (IF) and carbohydrate restriction/ketogenic diet (KD) are the most studied methods that are beneficial for cancer prevention.

CR prevents tumorigenesis by decreasing metabolic rate and oxidative damage [2]. The mechanism behind IF is relatively simple: it postpones tumor growth by starving tumors from glucose for a short period [6]. KD used to treat refractory seizures in children for decades is a diet regimen composed of low carbohydrates (usually less than 50 g/day), high fat and enough proteins. KD can restrict glucose for ATP production and energy derivation in cancer cells [6]–[8].

The present results chiefly originate from animal models, such as spontaneous model, chemical induced model, transgenic model and transplanted model [9]. Since human clinical trials of dietary restriction are extremely rare, it is urgent to review the existing achievements regarding the cancer preventive efficacy of dietary restriction in animal models. The present systematic review was conducted to discuss the findings from the most relevant and recent studies concerning the effects of dietary restriction regimens on cancer prevention.

## Methods

### Literature search and inclusion criteria

Keywords including “calorie restriction”, “caloric restriction “, “intermittent fasting”, “carbohydrate restriction”, “ketogenic diet”, “cancer” and “tumor” on Pubmed published between 1994 to January 2014 were searched, with limitation to English language. The inclusion criteria are: 1. studies on the anticancer effects of CR, IF or KD; 2. studies using animal models; 3. studies reporting at least one of the outcome measures associated with antitumor effects. Studies in vitro and on human participants were excluded. Repeated studies performed by the same author would not be included.

The titles and abstracts of the obtained articles were reviewed by two reviewers (M.M.L. and X.Y.Z.) independently. After excluding the articles not meeting the inclusion criteria, the two reviewers read the whole passage of the remaining articles to make sure they truly met the inclusion criteria. Any controversy was resolved by discussion with the third reviewer (H.W.) to reach consensus amongst all reviewers.

### Quality assessment and data extraction

Two reviewers (M.M.L. and X.Y.Z.) independently appraised each included article according to a critical checklist of the Stroke Therapy Academic Industry Roundtable (STAIR) [10]. The key points of this checklist include: 1. performing appropriate sample size calculations; 2. defining inclusion/exclusion criteria a priori; 3. reporting the generation of stochastic sequence; 4. providing the method of concealing random allocation sequence; 5. reporting the reasons for excluding animals from the final data analysis; 6. eliminating outcome assessment bias; 7. declaring relevant conflicts of interest.

Two reviewers (M.M.L. and X.Y.Z.) independently extracted data. Information including animal model, tumor type, feeding regimen, study length, sample size, major outcome, conclusion, quality assessment score and the interferential step of cancer was extracted from each study using a preset form.

### Data analysis

Data were analyzed on Stata 12 (Stata Corporation, College Station, Texas, USA). Dichotomous data were tested using odds ratio (OR) and its 95% confidence interval (CI). Heterogeneity was examined by Chi-square test [11]. A fixed-effects model was used if homogeneity was significant (P>0.1, I^2^<50%), and otherwise, a random-effects model was used.

## Results

### Eligible studies

**Figure 1:**
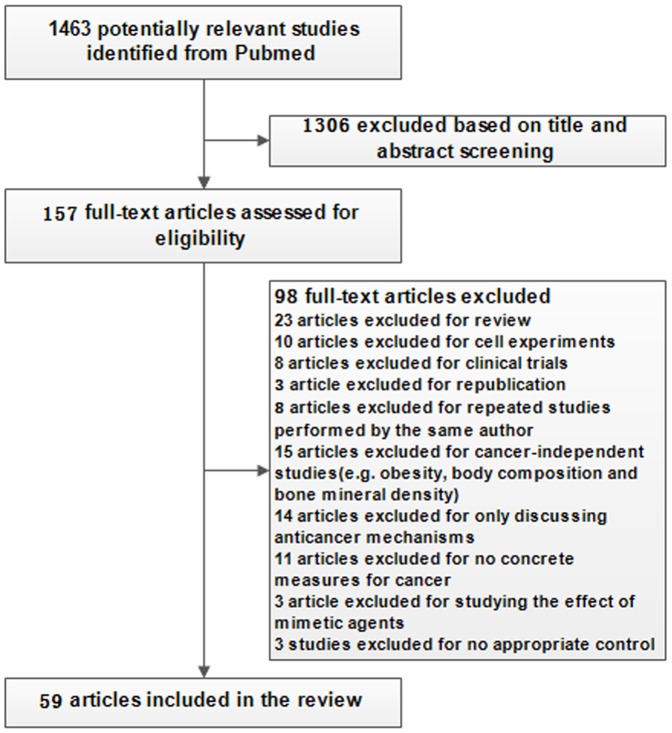
Flow chart of the selection of studies included in the system review.

The flow of search strategy is showed (Fig. 1). A total of 1463 articles were identified from Pubmed and 1306 studies were excluded after reviewing title and abstract, with a selection of 157 studies for detailed review. Twenty-three reviews, ten cell experiments, and eight clinical trials were subsequently excluded after full-text reading according to the inclusion criteria. Three republicated animal studies, and eight repeated studies performed by the same author were excluded. Fifteen cancer-irrelevant studies were excluded (e.g. obesity, body composition and bone mineral density). Fourteen studies only discussing anticancer mechanisms and eleven studies without providing concrete measures for cancer were also excluded. Three studies studying the effect of agents and three studies with no appropriate control were excluded.

Finally, a total of 59 animal studies fulfilled the inclusion criteria. The characteristics, major outcomes and methodological quality assessment results of each study are given in Table 1, 2, 3. All the included studies used cancer murine models, except for one study evaluating epithelial ovarian cancer (OVAC) preventive strategies which used the chicken model. Spontaneous model, chemical induced model, transgenic model and transplanted model were adopted by the included studies. Two types of hormone­sensitive cancers— breast cancer and prostate cancer were most studied, followed by brain cancer and hepatic cancer. The scores of qualities of the studies using STAIR ranged from 3 to 5.

**Table 1:** Animal experiments of caloric restriction diet and cancer.

Author(Year)	Model	Tumor	Feeding Regimens	Sample size	Time^a^	Body weights(g)	Major Results	C^b^	Q^c^	S^d^
Engelman 1994	Mice	Mammary, TG**^e^**	AL**^f^**;CR**^g^** (4–12w**^h^**); CR(continuously)	60;24;60	60	42.3; 41.4; 27.8	Tumor incidence(%): 83; 50; 13	+	4	I
Tagliaferro 1996	Rats	Mammary, C**^i^**	AL; Cyclic CR(1w 33% restriction 3w refeeding)	47;49	16	Cyclic CR<AL	Tumor incidence(%): 54; 66	-	4	I
Gillette 1997	Rats	Mammary, C	AL; 20%CR	30;30	20.5	CR<AL	Tumor incidence(%): 23.3; 6.7	+	3	I
Pape-Ansorge 2002	Mice	Mammary, TG	AL; ICR**^j^**(3 weeks 50% CR 3 weeks AL);CCR**^k^**	32;31;33	80	34.9; 31.1; 28.0	Tumor incidence(%): 37.5; 22.5; 33	+	4	I
Thompson 2004	Rats	Mammary, C	40% CR;AL	54;24	11	162;207	Tumor incidence(%): 59;96	+	4	I
Zhu2005	Rats	Mammary, C	40%CR; 6 week 40%CR 8 day refeeding; AL	30;20;29	7	139;160;191	Tumor incidence(%): 56.7;80;96.6	+	3	I
Cleary2007	Mice	Mammary, TG	ICR(3 weeks 50% CR 3 weeks AL);CCR; AL	39;30; 31	80	25/32.5**^l^**;26.2; 31.2	Tumor incidence(%): 15;27; 84	+	3	I
Jiang 2008	Rats	Mammary, C	20% CR; 40% CR;AL	30;30;30	>7	150;123;180	Tumor incidence(%): 60;23;96	+	4	I
Dogan 2009	Mice	Mammary, TG	ICR(3 weeks 50% CR 3 weeks AL); CCR;AL	52;40;44	64	22.6/26.7;25.1;36	Tumor incidence(%): 11.5;20; 45.5	+	5	I
Phoenix 2010	Mice	Mammary, TP**^m^**	30%CR;AL	/**^n^**	>27	/	Tumor volume: CR<AL; Metastases: CR<AL	+	3	P, M
De Lorenzo 2011	Mice	Mammary, TP	40%CR; Normal diet	7;7	9	16.6; 21.6	Wet tumor weight: 1.5; 3.5 g; Metastases: CR<AL	+	4	P, M
Nogueira 2012	Mice	Mammary, TP	30% CR; control diet	15;15	18	29;40	Tumor weight: 0.04;0.39 g	+	4	P
Dunlap 2012	Mice	Mammary, TP	30%CR;AL	20;20	>42	/	Tumor area: CR<AL	+	3	P
Saleh2013	Mice	Mammary, TP	ADF(alternate day feeing); 30%CR; AL	80(total)	6	CR<AL	Tumor growth delay of ADF and CR	+	4	P
Mizuno 2013	Mice	Mammary, TG	CCR; ICR(3weeks 50% CR 3weeks AL); AL	36;29;30	>50	CR<AL	Tumor incidence(%): 47; 59; 87	+	4	I
Rogozina 2013	Mice	Mammary, TG	ICR(3 weeks 50%CR 3 weeks AL); CCR; AL	45;45;45	82	CR<AL	Tumor incidence(%): 4.4;52.3; 66.7	+	4	I
Boileau 2003	Rats	Prostate, C	AL; 20%CR	194 total	>60	CR<AL	Prostate cancer-free survival: CR>AL	+	4	I
SUTTIE 2005	Mice	Prostate, TG	Late-onset 20%CR**^o^**; AL	109(total)	39	CR<AL (sex-pluck)	CR retard epithelial lesion development	+	3	P
Kandori 2005	Rats	Prostate, TG	30%CR; control	10;10	91	389.3; 475.2	Decreased epithelial areas/whole area in CR	+	4	I
McCormick2007	Rats	Prostate, C	30%CR; 15%CR;AL	43;42;43	48	CR<AL	Tumor incidence(%):72;64;74	-	4	I
Bonorden2009	Mice	Prostate, TG	ICR(2 weeks 50% CR 2 weeks AL);CCR; AL	101;79;41	50	27.43/30.89**^p^**;29.16; 33.48	Median time to tumor detection (week): 38;35; 33	+	4	I
Blando 2011	Mice	Prostate, TG	30%CR;overweight control; diet-induced obesity	27;23;23	24	23.9;40.1;44.9	Tumor incidence(%):37;100;100	+	4	I
Galet 2013	Mice	Prostate, TP	40% CR; AL	16;16	>3	CR<AL	Tumor weight:295; 467 mg	+	4	P
Seyfried 2003	Mice	Brain, TP	AL; 40%CR	7;6	>2	CR<AL	Tumor dry weight: CR<AL	+	3	P
Shelton2010	Mice	Brain, TP	60%CR;AL	9-10;9-10	>2	CR<AL	CR reduced the growth and invasion of tumor	+	4	P, M
Mulrooney 2011	Mice	Brain, TP	30%CR; AL	5; 4	>14	CR<AL	Tumor weight: CR<AL	+	4	P
Jiang 2013	Mice	Brain, TP	40%CR;AL	30;30	>14	CR<AL	Tumor weight: CR<AL	+	3	P
Birt 1997	Hamster	Pancreatic, C	AL; 10%CR; 20%CR; 40%CR	35;35;38;33	102	CR<AL	Tumor incidence: 14;9;13;18	-	4	I
Lashinger 2011	Mice	Pancreatic, TP	30%CR; AL	9;9	11	CR<AL	Tumor weight: CR<AL	+	4	P
Lanza-Jacoby2013	Mice	Pancreatic, TG	ICR (1 week 50% CR 1week AL);CCR; AL	31;31;31	44	21.7;21;29.6	Incidence of PanIN-2 or more lesions: 27;40; 70%	+	5	I
James 1994	Mice	Hepatic, S**^q^**	AL; 40%CR	73;72	144	32.3; 23.5	Tumor incidence(%): 27.4; 4.2	+	4	I
Von Tungeln,1996	Mice	Hepatic, C	AL; 40%CR	46; 42	84	CR<AL	Tumor incidence(%): 41.3; 0	+	4	I
Van Ginhoven 2010	Mice	Hepatic, TP	30%CR(preoperative);AL	5;5	24	CR<AL	Hepatic tumor load: reduced by CR	+	3	P
Stewart 2005	Mice	Skin, C	40%CR; AL	32;30	>31	CR<AL	Papilloma incidence: CR<AL	+	3	I
Moore 2012	Mice	Skin, C	30% CR; 15% CR;10 kcal% fat; 60 kcal% fat	26;29;27;25	>50	26.7;35.0;41.4;50	Tumor incidence(%):57.7;69;92.3;96	+	4	I
Tomita 2012	Rats	Colonic, C	40%CR; AL	23;23	5	CR<AL	Number of aberrant crypt foci: CR<AL	+	4	I
Harvey 2012	Mice	Colonic, TP	30%CR; AL	30;30	>24	CR<AL	Tumor volume: CR<AL	+	4	P
Carver 2011	Bird	Ovarian, S	55%CR; full-fed	394;393	2year	1423;1896	Tumor incidence(%):10.3;33.3	+	4	I
Mai 2003	Mice	Intestinal, TG	AL; 40%CR	30;28	9	CR<AL	Polyp numbers: CR<AL	+	3	I
Dunn 1997	Mice	/, TG+C	AL; 20%CR	10;10	22	38; 30	Tumor incidence(%): 40; 20	+	3	I
Hursting, 1997	Mice	/, S	AL(P53-);40%CR(p53-); AL(p53+); 40%CR(p53+)	28-30/group	132	CR<AL	CR delayed tumor mortality relative to AL	+	4	I
Berrigan 2002	Mice	/, TG	AL; 40%CR; 1day/week fast	31-32/group	>48	CR<Fast<AL	Tumor free survival: CR>AL; Fast>AL	+	4	I
Tsao 2002	Mice	/, TG	Control; High fat/low calcium; 30%CR	34;46;16	/	CR<Control	Intestinal tumor incidence(%): 68; 65; 69	-	3	I
Yamaza2010	Mice	/, TG	30%CR; AL	18;17	>144	CR<AL	Tumor incidence(%): 16.7; 94.1	+	3	I

a Time: Time of study (weeks); **^b^**C: Conclusion of the study, “+” indicates a positive conclusion and “-” represents a negative conclusion; **^c^**Q: Quality of the study according to a critical checklist of the Stroke Therapy Academic Industry Roundtable; **^d^**S: The step(s) of cancer that dietary restriction regimens interfere during the initiation, progression and metastasis of cancer, “I” indicates initiation, “P” indicates progression and “M” indicates metastasis; **^e^**TG: transgenic; **^f^**AL: Ad libitum; **^g^**CR: caloric restriction; **^h^**w: week; **^i^**C: Chemical-induced; **^j^**ICR: Intermittent caloric restriction; **^k^**CCR: chronic caloric restriction; **^l^**25/32.5: ICR mice sacrificed at the end of the 12^th^ restriction period/ICR mice sacrificed at 1week after 12th refeeding; **^m^**TP: transplanted; **^n^**/: not specified; **^o^**Late-onset 20%CR: al libitum 20 weeks followed by 20% diet restriction; **^p^**27.43/30.89: Mice euthanized during restriction/Mice euthanized during AL consumption; **^q^**S: Spontaneous.

**Table 2:** Animal experiments of carbohydrate restriction/ketogenic diet and cancer.

Author(Year)	Model	Tumor	Feeding Regimens	Sample size	Time^a^	Body weights(g)	Major Results	C^b^	Q^c^	S^d^
Zhou 2007	Mice	Brain, TP**^e^**	High-C**^f^**; KC **^g^**; KC-R(KC-restricted)	18;16;25	>6	Lower in KC-R group	Tumor wet weight: KC-R<high C.	+	4	P
Stafford 2010	Mice	Brain, TP	SD**^h^**; KD**^i^**	20	>4	/**^j^**	Tumor growth: KD <SD	+	3	P
Abdelwahab2012	Mice	Brain, TP	SD;KC; SD+Radiation; KC+Radiation	19;19;11;11	>40	/	KC enhances anti-tumor effect of radiation	+	4	P
Freedland 2008	Mice	Prostate, TP	NCKD**^k^**; low-fat; Western diet	25;25;25	>10	Reduced in NCKD	Tumor volumes: NCKD<Western Diet	+	5	P
Mavropoulos2009	Mice	Prostate, TP	NCKD;low fat/high-C(LFD);high-fat/moderate-C(MCD)	48;41;41	18	Maintained finally	Tumor volume: LFD<MCD; NCKD<MCD	+	5	P
Wheatley2008	Mice	Colonic, TP	low-C; high-C(HC); HC restricted; diet-induced obesity	20;20;20;20	23	Less in HC,HCrestricted	Tumor size:351.6;474.6;162.4; 397.2 mm^2^	-	4	P
Otto 2008	Mice	Gastric, TP	SD; KD	12;12	>6	29.9;29.6	Tumor growth: KD <SD	+	4	P
Poff 2013	Mice	Metastatic, TP	SD; KD	13;8	>3	Reduced weight of KD	Tumor growth: KD <SD	+	4	M
Ho 2011	Mice	/, TP	Western diet;8% C;15% C; 10%C	31;8;17;5	>3	8%,10%C<western diet;	Tumors growth: low C <western diet	+	3	P

a Time: Time of study (weeks); **^b^**C: Conclusion of the study, “+” indicates a positive conclusion and “-” represents a negative conclusion; **^c^**Q: Quality of the study according to a critical checklist of the Stroke Therapy Academic Industry Roundtable; **^d^**S: The step(s) of cancer that dietary restriction regimens interfere during the initiation, progression and metastasis of cancer, “I” indicates initiation, “P” indicates progression and “M” indicates metastasis; **^e^**TP: transplanted; **^f^**C: carbohydrate; **^g^** KC: a nutritionally balanced and commercially available ketogenic diet; **^h^** SD: standard diet; **^i^** KD: ketogenic diet; **^j^**/: not specified; **^k^**NCKD: no-carbohydrate ketogenic diet.

**Table 3:** Animal experiments of intermittent fasting and cancer.

Author(Year)	Model	Tumor	Feeding Regimens	Sample size	Time^a^	Body weights(g)	Major Results	C^b^	Q^c^	S^d^
BuschemeyerIII 2010	Mice	Prostate, TP**^e^**	AL; 1D**^f^**fasted 6D AL**^g^**; 1Dfasted 6D paired feeding; 14% CR**^h^**; 2Dfasted 5D AL; 2Dfasted 5D paired feeding; 28% CR	15/group	>5	Reduced body weights in the latter two groups	Tumor volume and survival: no significant differences.	-	4	P
Thomas II 2010	Mice	Prostate, TP	AL; IF (twice-weekly 24 h fasts)	50;50	>4	No significant difference	IF didn't delay tumor growth	-	4	P
Tomasi 1999	Rats	Hepatic, C	Control; IF (3D followed by 11D refeeding)	11;11	48	371; 368	Tumor incidence: 36%; 72%	-	4	I
Rocha 2002	Rat	Hepatic, C**^i^**	AL; IF (48 h weekly fasting)	12;12	52	355.2; 445.8	Number, size of liver nodules: IF<AL	+	4	I
Saleh2013	Mice	Mammary, TP	IF(alternate day feeing); 30%CR; AL	80(total)	6	Reduced weight in CR	Tumor growth delay of ADF and CR	+	4	P
Lee 2012	Mice	Multiple, TP	Control, two cycles of fasting(48 h each)	41(total)	>6	Regain weight when refeeding	Fasting retard tumor growth	+	3	P
Marsh 2008	Mice	Brain, TP	Late-onset intermittent CR feeding; AL	7;8	>20	Reduced in intermittent feeding	Tumor weight: IF<AL	+	3	P
Berrigan 2002	Mice	/, TG**^j^**	AL; 40%CR;IF(1day/week)	31-32/group	>48	CR<Fast<AL	Tumor free survival: CR>AL; Fast>AL	+	4	I

a Time: Time of study (weeks); **^b^**C: Conclusion of the study, “+” indicates a positive conclusion and “-” represents a negative conclusion; **^c^**Q: Quality of the study according to a critical checklist of the Stroke Therapy Academic Industry Roundtable; **^d^**S: The step(s) of cancer that dietary restriction regimens interfere during the initiation, progression and metastasis of cancer, “I” indicates initiation, “P” indicates progression and “M” indicates metastasis; **^e^**TP: transplanted; **^f^**D: Day; **^g^**AL: Ad libitum; **^h^**CR: caloric restriction; **^i^**C: Chemical-induced; **^j^**TG: transgenic.

### CR

Forty-four included studies that evaluated antitumor effects in animals were placed on CR [12]–[55] (Table 1). Among them, murine models were most frequently used (43 studies) and chicken model was used in one study. The most studied cancer types were mammary, prostate, brain, pancreatic, and hepatic cancers. Skin, colonic, ovarian and intestinal cancers were also investigated each in one or two related studies. Spontaneous model, chemical induced model, transgenic model and transplanted model were applied. Forty of the forty-four studies (90.9%) supported the positive anticancer role of CR despite the different measurements. Thirty studies investigated the role of CR on initiation of cancer, twenty-six of which addressed the preventive role of CR on cancer initiation. Fourteen studies explored the effect of CR on progression and three of them were also on metastasis of cancer, all of these studies showed that CR modulated progression and metastasis of cancer. The most used measurement was tumor incidence expressed in percentage. Tumor growth, tumor weight and other measurements were also applied. From the included studies, CR tended to be associated with reduced weight comparing to the controls.

Intermittent caloric restriction (ICR) and chronic caloric restriction (CCR) were studied separately by seven studies [13], [15], [17], [24], [27], [32], [51]. The period of restriction ranged from one week to three weeks in ICR, followed by an equal time of feeding at AL. Six of the seven studies concluded clearly that ICR was more effective in tumor prevention than CCR, while the remaining study did not specify (data not shown).

Moreover, one study showed that late-onset CR which means applying CR diet after a period of AL diet also retarded epithelial lesion development.

### KD

Nine studies explored the relationship between carbohydrate restriction and cancer [56]–[64] (Table 2). All studies used the murine models. The studied tumors included prostate, brain, colonic, gastric and metastatic cancers. Transplanted models were applied by all the involved studies. Eight of the nine studies (88.9%) supported that carbohydrate restriction is protective on cancer. One study using the mice model and colon cancer showed that low carbohydrate diet could not slow down tumor growth. Eight articles investigated the role of KD on progression of cancer, and seven of them held a positive conclusion. One article researched the role of KD on metastasis of cancer and indicated the role is efficient. Weight changes were not uniform among the involved studies. The composition of carbohydrate in the studies ranged from 0 to 20%. The major results were presented as tumor growth and tumor volume. A nutritionally complete and commercially available ketogenic diet was studied, and the two relevant studies all got positive conclusions although one was based on restricted amounts.

### IF

There are eight studies about IF and cancer [33], [42], [65]–[70] (Table 3). The fasting time ranged from 24 to 72 hours. The murine models were used. The most studied tumor types were prostate and hepatic cancers. Transplanted model, chemical-induced model and transgenic model were applied. Five of the eight studies (62.5%) got positive conclusion, two of them used fasting cycle (48 h) with no specified intermittent time and late-onset intermittent fasting. Three studies investigated the role of IF on initiation of cancer, and two of them showed the efficient role of IF. Five studies searched the role of IF on progression of cancer, and three of them supported the positive conclusion. Two studies analyzed both IF and CR, and IF was functional in delaying tumor growth although the effect was not obvious as CR. Three studies obtained a negative conclusion that IF was not significantly protective on cancer. The weight changes were not uniform among the involved studies.

### Meta-analysis

**Figure 2:**
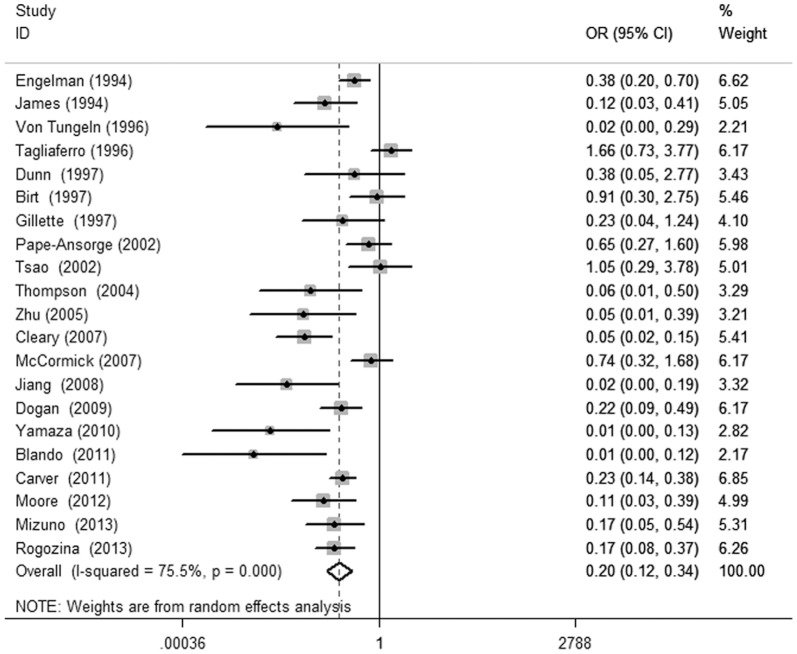
Forest plot for the association between caloric restriction diet and tumor incidence. Statistical analyse was performed using STATA (version 12), combined overall odds ratio (OR) was calculated using the random-effect model as heterogeneity existed (I2 = 75.5%, p<0.01).

Tumor incidence was the most frequently used outcome with specific data (in 22 studies). Twenty-one of them were about CR. The raw data of each study with tumor incidence were pooled in our study (Fig. 2). The random-effect model was applied as heterogeneity existed (I2 = 75.5%, p<0.01). The pooled OR (95%CI) for CR was 0.20 (0.12, 0.34) relative to the controls, and this indicated that CR plays a preventive role against cancer.

## Discussion

In this study, we reviewed the 59 animal experimental studies on dietary restriction regimens and analyzed the data to study roles of caloric restriction, ketogenic diet and intermittent fasting during initiation, progression and metastasis of cancer in animal models. Our research indicates that CR is preventive on cancers as about 91% of relevant studies support the conclusion and the result of meta-analysis is significant. Our findings also indicate that KD can prevent cancer although there are no convincing pooled data. However, no enough evidence indicates the preventive effect of IF on cancers.

A meta-analysis on CR and spontaneous breast cancers in mice between 1942 and 1994 [71] found that energy-restricted animals developed 55% less breast cancers than the controls, which was similar to our findings focused on studies between 1994 and 2014.

Though CR was strongly associated with reduced cancer risk in animal models, the effect in human is still unknown. It is almost impossible to assess the long-term cancer incidence of healthy people with CR diet. The existing clinical trials were most conducted in obese cancer patients, with biomarkers as the most detected index. However, conclusions of these clinical trials were not always the same. In a study investigating the effect of dietary intervention, the newly-diagnosed obese prostate cancer patients were randomized to a CR diet group or a control group and differences in weight loss and insulin-like growth factor (IGF)-binding proterin-3 (IGFBP-3) levels were found in the CR group [72]. IGFBP-3 is the most abundant IGFBP and serum level is positively associated with prostate cancer [73], [74]. In a study about obese postmenopausal women, no significant changes of IGF-1 or IGFBP-3 were detected in the dietary-induced weight loss group, but the ratio of IGF-1/IGFBP-3 increased in this intervention group [75], which were inconsistent with another study [72] or with the findings from animal experiments [24], [27]. In a randomized controlled trial, the levels of inflammation biomarkers were reduced in postmenopausal women with a CR weight loss diet [76], and this result was meaningful as increased levels of inflammatory biomarkers are associated with increased risk for some cancers [77]–[79]. Gene expression in breast tissue was also studied in obese women, as well as abdominal tissues, and significant changes were detected in glycolytic and lipid synthesis pathways following CR [80]. And gene included like Stearoyl-CoA desaturase (SCD) was found to be a key factor in regulation of tumorigenesis in vivo [81].

The included animal experiments indicate that ICR is more effective than CCR in prevention of cancers. A clinical trial [82] comparing ICR (2 days/week) and CCR in young overweight women showed that both ICR and CCR involved a 25% energy restriction. Except that ICR was equally effective for weight loss as CCR, the changes of many markers detected like CPR, IGF-1, IGFBP-1, and IGPBP-2 were also similar between the two groups. The study obtained a conclusion that ICR may be an equivalent alternative to CCR for weight loss and reducing disease risk.

KD may also have great potential in cancer prevention in our study, which was supported by eight of the nine included studies. The relationship between KD and cancer is unclear in the clinical realm. One study [83] comparing the effect of intermittent energy and carbohydrate restriction (<40 g carbohydrate/d for 2 d/week) with daily energy restriction in overweight women showed that the former is superior to the latter in improvement of insulin sensitivity and reduced body fat. However, this study was not directly related to KD. Nebeling et al [84] tried to assess the effects of ketogenic diet in two patients with advanced malignant astrocytoma tumors, the result that glucose uptake at the tumor site was reduced. Several existing clinical trials detecting KD in the oncology population are still ongoing [6].

IF may not be an ideal dietary intervention in animal experiments since 37.5% of the included studies provided negative results. However, the results of clinical experiments are unclear. A case series report [85] showed that fasting combined with chemotherapy is safe and may weaken the chemotherapy-induced side effects although only 10 cases were included. In the research, patients voluntarily fasted for up to 180 hours before and/or following chemotherapy [85]. Fasting cycles combined with chemotherapy drugs were also studied in animal experiments [66], and were effective and could prolong cancer-free survival. However, clinical data for IF are sparse, and some other existing clinical trials assessing IF in the oncology population are still carried on [6].

However, human experience for applying these dietary restriction regimens in cancer prevention is limited. There are many shortcomings in the existing clinical experiments. Firstly, many studies lack control groups and reliabilities of these studies are not enough. Secondly, the restriction regimens cannot always be tolerated by all the subjects through the study. Thirdly, the research periods are short, and the long-term effects of dietary regimens cannot be well explained. Fourthly, the results are often shown as changes of biomarkers instead of direct evidence.

Moreover, there are several obstacles on the way to use these dietary restriction regimens as a treatment or preventive intervention for cancer. For example, some dietary intervention methods are unadherable in the long run. Many side effects can be caused [6]. However, researchers are trying to solve the challenges so as to adopt these dietary habits into humans. For example, an effective promoted way is CR mimetics, which can also play an anticancer role like CR but without requiring drastic energy restriction [86]. IGF-1 and Akt/mTOR pathways are potential important mediators in the anticancer function of CR, and pharmacologic interventions targeted at these pathways are of great value. A variety of agents will affect the pathways [87]. Some agents targeting at IGF-1 receptor like monoclonal antibodies and small-molecule tyrosine kinase inhibitors are under clinical trials for many cancers [88].

Prospectively, the role of dietary restriction regimens against cancers in animal models has been studied extensively, but the achievements have not been verified in humans. Therefore, more clinical experiments are needed. Regarding the difficulty in applying these dietary restrictions into humans, more tolerable regimens should be developed. Since conditions differ among cancer patients, individualized treatment plan is necessary, so that each patient can achieve the best therapeutic effect. The incidence of malnutrition is high in cancer patients, and some patients even suffer from cachexia. Consequently, dietary restriction therapy might be a problem for these patients as nutritional support is necessary. There should be a balance between dietary restriction and nutritional support. Efforts should be made to thoroughly investigate the mechanism of dietary regimens acting on tumors, and develop agents interfering with the pathways. Mimetics which can replace dietary modifications is a progressing potential area.

In this study, we reviewed animal experimental data of three dietary restriction regimens (CR, IF and KD) and pooled the accessible tumor incidence data of CR. This study has some limitations. First, only experiments since 1994 were collected, which may affect our conclusions because there are also some valuable studies before. Second, heterogeneity existed when pooling the data of CR, probably due to the differences in animal models, cancer types, sample size, or observation time. Third, other data such as tumor volume and survival time were not pooled due to the small number of relevant studies. Fourth, there are few clinical experiments, thus only animal experiments were systematically analyzed.

In conclusion, the research indicates that CR and KD are effective in prevention of cancers in animal experiments, but the role of IF is doubtful. More clinical trials are needed to investigate the effectiveness and safety of these dietary regimens. Dietary restriction regimen which is more suitable in human for cancer prevention and therapy should be detected. And the valuable but more tolerable ways that can replace dietary restriction should be further explored.

## Supporting Information

S1 Checklist PRISMA Checklist of this meta-analysis.
(DOC)
